# Adherence and feasibility of 2 treatment schedules of S-1 as adjuvant chemotherapy for patients with completely resected advanced lung cancer: a multicenter randomized controlled trial

**DOI:** 10.1186/s12885-017-3584-y

**Published:** 2017-08-29

**Authors:** Yoshinobu Hata, Takaharu Kiribayashi, Kazuma Kishi, Makoto Nagashima, Takefumi Nakayama, Shingo Ikeda, Mitsutaka Kadokura, Yuichi Ozeki, Hajime Otsuka, Yoshitaka Murakami, Keigo Takagi, Akira Iyoda

**Affiliations:** 1 Division of Chest Surgery (Omori), Toho University School of Medicine, 6-11-1 Omori-nishi, Ota-ku, Tokyo, 143-8541 Japan; 2 Division of Chest Surgery (Ohashi), Toho University School of Medicine, 2-17-6 Ohashi, Meguro-ku, Tokyo, 153-8515 Japan; 3 Department of Respiratory Medicine, Respiratory Center, Toranomon Hospital, 2-2-2 Toranomon, Minato-ku, Tokyo, 105-8470 Japan; 4 Division of Chest Surgery (Sakura), Toho University School of Medicine, 564-1 Shimosizu, Sakura, Chiba, 285-8741 Japan; 5 Department of Thoracic and Cardiovascular Surgery, Japan Self-Defense Forces Central Hospital, 1-2-24 Ikejiri, Setagaya-ku, Tokyo, 154-8532 Japan; 6 Department of Thoracic Surgery, Mitsui Memorial Hospital, 1 Kandaizumicho, Chiyoda-ku, Tokyo, 101-8643 Japan; 7 Division of Chest Surgery, Department of Surgery, Showa University School of Medicine, 1-5-8 Hatanodai, Shinagawa-ku, Tokyo, 142-8555 Japan; 8 Department of Thoracic Surgery, National Defense Medical College, 3-2 Namiki, Tokorozawa, Saitama, 359-0042 Japan; 9 Department of Medical Statistics, Toho University School of Medicine, 5-21-16 Omori-nishi, Ota-ku, Tokyo, 143-8540 Japan

**Keywords:** Lung cancer, Adjuvant chemotherapy, S-1

## Abstract

**Background:**

We conducted a multicenter randomized study of adjuvant S-1 administration schedules for surgically treated pathological stage IB-IIIA non-small cell lung cancer patients.

**Methods:**

Patients receiving curative surgical resection were centrally randomized to arm A (4 weeks of oral S-1 and a 2-week rest over 12 months) or arm B (2 weeks of S-1 and a 1-week rest over 12 months). The primary endpoints were completion of the scheduled adjuvant chemotherapy over 12 months, and the secondary endpoints were relative total administration dose, toxicity, and 3-year disease-free survival.

**Results:**

From April 2005 to January 2012, 80 patients were enrolled, of whom 78 patients were eligible and assessable. The planned S-1 administration over 12 months was accomplished to 28 patients in 38 arm A patients (73.7%) and to 18 patients in 40 arm B patients (45.0%, *p* = 0.01). The average relative dose intensity was 77.2% for arm A and 58.4% for arm B (*p* = 0.01). Drug-related grade 3 adverse events were recorded for 11% of arm A and 5% of arm B (*p* = 0.43). Grade 1–3 elevation of bilirubin, alkaline phosphatase, aspartate aminotransferase, and alanine transaminase were more frequently recorded in arm A than in arm B. The 3-year disease-free survival rate was 79.0% for arm A and 79.3% for arm B (*p* = 0.94).

**Conclusions:**

The superiority of feasibility of the shorter schedule was not recognized in the present study. The conventional schedule showed higher completion rates over 12 months (*p* = 0.01) and relative dose intensity of S-1 (*p* = 0.01). Toxicity showed no significant difference among the shorter schedule and the conventional schedule, except for grade 1–3 elevation of bilirubin.

**Trial registration:**

This randomized multicenter study was retrospectively registered with the UMIN-CTR (UMIN000016086, registration date December 30, 2014).

## Background

Lung cancer is the leading cause of cancer-related death worldwide [1]. During the last decade, adjuvant cisplatin-based chemotherapy has become the standard therapy for patients with completely resected stage IIA to IIIA non–small cell lung cancer (NSCLC) [2]. The pooled Lung Adjuvant Cisplatin Evaluation (LACE) study [3] confirmed that adjuvant chemotherapy achieved a survival benefit of approximately 5% at 5 years. The Japan Lung Cancer Research Group (JLCRG) trial [4] has shown that postoperative tegafur-uracil (UFT; Taiho Pharmaceutical Co., Ltd., Tokyo, Japan) can improve the survival of completely resected stage I lung adenocarcinoma patients, providing a significant overall survival advantage of 11% at 5 years for patients with T2 disease. A meta-analysis of UFT as postoperative adjuvant chemotherapy [5] for NSCLC showed that survival rates at 5 years were significantly higher in patients who received UFT after surgery than in those who underwent surgery only (82% vs 77%; respectively). A recent analysis reported an overall survival advantage of 6% at 5 years for patients with T1b NSCLC who received UFT [6], and postoperative adjuvant UFT for 1 or 2 years has become the standard therapy for patients with completely resected stage IA (> 2 cm) and IB NSCLC in Japan.

S-1 (Taiho Pharmaceutical Co., Ltd., Tokyo, Japan) is a second-generation oral fluoropyrimidine composed of tegafur, gimeracil, and oteracil in a molar ratio of 1:0.4:1 [7]. Postoperative adjuvant chemotherapy with S-1 has shown significant survival benefit for patients with gastric cancer [8], and S-1 is expected to be a promising agent for use in an adjuvant setting, with higher antitumor activity than UFT. S-1 has been conventionally prescribed as an oral agent that is administered twice daily for 4 weeks followed by a 2-week rest period. A treatment schedule that shortened the conventional schedule by half (2-weeks of administration followed by a 1-week rest) was reported to be more feasible for patients with advanced head and neck cancer who had undergone definitive treatment [9]. While the shorter administration schedule was expected to be more feasible for definitively treated lung cancer patients, the completion rates of adjuvant S-1 administration for patients with completely resected lung cancer have been reported to be 61%–71% for 6 months with the shorter schedule [10, 11] and 50%–72% for 1-year with the conventional schedule [12, 13]. The optimal administration schedule of S-1 in the adjuvant setting for patients with completely resected NSCLC has not yet been investigated.

We therefore performed a multicenter randomized phase II study, comparing the feasibility of the conventional treatment schedule of S-1 administered for 4 weeks followed by a 2-week rest and the shorter treatment schedule of S-1 administered for 2 weeks followed by a 1-week rest, as adjuvant treatment of patients with completely resected NSCLC.

## Methods

### Eligibility criteria

The criteria for eligibility were as follows: histologically confirmed primary lung adenocarcinoma, squamous cell carcinoma, large cell carcinoma, and adenosquamous carcinoma; complete resection of the primary tumor (R0 resection); pathological stage IB to IIIA disease (TNM version 6); patients aged 20 to 74 years; Eastern Cooperative Oncology Group (ECOG) performance status (PS) of 0 or 1; and adequate organ function (leukocyte count of at least 4000 mm^3^, absolute neutrophil count of at least 2000 mm^3^, platelet count of at least 100,000 mm^3^, hemoglobin level of at least 9.0 g/dL, aspartate aminotransferase [AST] and alanine transaminase [ALT] levels lower than 2.5-fold the upper limit of normal, total bilirubin level of 1.5 mg/dL or less, creatinine level lower than the upper limit of normal, 24-h creatinine clearance rate of higher than 50 mL/min); able to start within 9 weeks after surgery; and no prior therapy.

The exclusion criteria were as follows: history of previous chemotherapy, radiotherapy or surgery for lung cancer; pulmonary fibrosis; pleural effusion, ascites, or cardiac effusion that required drainage; concomitant malignancy; significant comorbidity (poorly controlled angina, myocardial infarction within 3 months, cardiac failure, poorly controlled diabetes mellitus, severe infection, and others); diarrhea; pregnancy; desiring to have children; and drug allergy to S-1 or any of its components.

The study protocol was approved by the local ethics committee at each participating center. All patients provided written informed consent to participate.

### Study design and treatment

The primary endpoints were the rates of completing the planned administration schedule over 12 months; the secondary endpoints were relative total administration dose of S-1, toxicity, and 3-year disease-free survival (DFS). The completion rates over 12 months were calculated regardless of the presence or absence of dose reduction. The relative total administration dose (relative dose intensity) was defined as (the actual total dose administered divided by the planned total administered dose) × 100. Feasibility was evaluated by the completion rates over 12 months and the relative dose intensity of S-1. Patient randomization was performed centrally at the Division of Chest Surgery, Toho University School of Medicine, Tokyo, Japan, with the following stratification factors: pathological stage, histology and gender. Sample size was set to 40 patients to each group, based on the feasibility. S-1 was administered orally after meals. The dosage of S-1 was selected as follows: for a patient with a body surface area (BSA) < 1.25 m^2^, 40 mg twice a day (80 mg/day); BSA of 1.25 m^2^ or more but <1.5m^2^, 50 mg twice a day (100 mg/day); and BSA of 1.5 m^2^ or more, 60 mg twice a day (120 mg/day). Patients who underwent complete resection were randomly assigned to either arm A, S-1 administration for 4 weeks followed by a 2-week rest period; or arm B, S-1 administration for 2 weeks followed by a 1-week rest. For both treatment arms, the administration of S-1 was continued for 12 months (8 courses for arm A and 16 courses for arm B), unless there was any evidence of recurrence, other malignancies, or severe adverse events. Lafutidine, a histamine H2 receptor antagonist, was administered at 10 mg twice a day to all the patients to reduce gastrointestinal toxicities. The patient’s visit was planned at least every 6 weeks just before the initiation of each course in A arm or each even course in B arm.

During the study, the dosage of S-1 was adjusted according to the degree of toxicities. The planned dose reduction was from 120 mg to 100 mg, 100 mg to 80 mg, or 80 mg to 50 mg, for patients with evidence of grade 3 hematological toxicity (except for thrombocytopenia), grade 2 thrombocytopenia, grade 2 or higher nonhematological toxicity (except for renal dysfunction), or grade 1 renal dysfunction. If a patient receiving a reduced dose of 50 mg/day continued to manifest or redeveloped toxicity as described, then treatment with S-1 was stopped.

### Evaluations of feasibility and toxicity

Feasibility and toxicity analyses were conducted on the intent-to-treat principle, which included all the patients in the randomization. Feasibility was evaluated by the completion rates over 12 months and the relative dose intensity. The number of patients in each arm was calculated at the time when S-1 administration was reduced or stopped because of any reasons including an adverse event associated with S-1, patient refusal, tumor recurrence or other non-S-1-related complication.

The planned duration of follow up of each patient in each arm was 3 years after randomization. Adverse events were assessed according to the National Cancer Institute-Common Terminology Criteria for Adverse Events v3.0 (CTCAE).

### Statistical analysis

Our purpose of this study is to compare the feasibility and toxicity of the short treatment schedule of S-1 (2 week administration with 1 week rest) and the conventional one (4 week administration with 2 week rest). These comparisons were conducted by the completion rates over 12 months, relative dose intensity of S-1, and toxicity. Patient characteristics, feasibility, adverse events, and disease-free survival were analyzed. The completion rates over 12 months were compared by the chi-square test. The difference in mean values of the relative dose intensity of the 2 arms was evaluated using the Student t-test. The rates of adverse events were compared by the chi-square test. Three-year DFS were estimated using the Kaplan–Meier method, and differences between the 2 arms were examined using the log-rank test. The level of significance was set at *p* = 0.05.

## Results

### Patient characteristics

From April 2005 to January 2012, 80 patients with stage IB to IIIA NSCLC who had undergone complete resection were enrolled and randomized centrally (39 cases to arm A and 41 to arm B). After randomization, 2 patients were found to be ineligible. One arm A patient was histologically diagnosed with pleomorphic carcinoma, and 1 arm B patient was diagnosed with stage IIIB disease (pT4 with intrapulmonary metastasis). The number of patients in the intent-to-treat analysis was 38 cases in arm A and 40 in arm B. The groups were well balanced with regard to baseline clinical characteristics, surgical procedures, and histopathological findings (Table 1). Adenocarcinoma was the most frequent histological subtype, occurring in 68% of arm A and 60% of arm B patients. Pathological stage IB disease was confirmed in 74% of arm A and 83% of arm B patients. The surgical procedure was lobectomy with mediastinal lymph node resection in 100% of arm A and 93% of arm B patients. Three arm B cases (8%) underwent bilobectomy, pneumonectomy, or segmentectomy with mediastinal lymph node resection.

**Table 1:** Patent characteristics

Characteristics	Arm A (*n* = 38)		Arm B (*n* = 40)		*p*
Male:female, *n* (%)	26 (68): 12 (32)		25 (63): 15 (37)		0.58
Mean (SD) age, years	62	(6)	63	(9)	0.39
Mean (SD) BSA, m^2^	1.59	(0.15)	1.61	(0.18)	0.72
PS, *n* (%)					
0	36	(95)	37	(92)	1.00
1	2	(5)	3	(8)	
Histology, *n* (%)					
Adenocarcinoma	26	(68)	24	(60)	0.70
Squamous cell ca.	10	(26)	13	(32)	
Large cell carcinoma	2	(5)	2	(5)	
Adenosquamous ca.	0	(0)	1	(3)	
Stage, *n* (%)					
IB	28	(74)	33	(82)	0.39
IIA	2	(5)	0	(0)	
IIB	5	(13)	3	(8)	
IIIA	3	(8)	4	(10)	
Surgical procedure, *n* (%)					
Lobectomy	38	(100)	37	(92)	0.40
Bilobectomy	0	(0)	1	(3)	
Pneumonectomy	0	(0)	1	(3)	
Segmentectomy	0	(0)	1	(3)	

*BSA* body surface area, *PS* performance status, *squamous cell* ca. squamous cell carcinoma, *adenosquamous* ca. adenosquamous carcinoma; *p* values for sex, PS, type of histology, pathological stage, and surgical procedure were calculated with the use of the chi-square test. *p* values for age and BSA were calculated with the use of the Student t-test

### Feasibility

The completion rates over 12 months were 73.7% (95% confidence interval [CI] 58.0%–85.0%) in arm A and 45.0% (95% CI: 30.7%–60.2%) in arm B patients (*p* = 0.01, Tables 2 and 3). Twenty-eight patients (73.7%) in arm A (12 patients with dose reduction) and 18 patients (45.0%) in arm B (3 patients with dose reduction, and 3 patients with delayed courses) received S-1 administration according the planned schedule. S-1 administration was halted because of adverse events or refusal for 7 (18%) of arm A (*n* = 6 adverse events, *n =* 1 refusal) and 15 (38%) of arm B patients (*n* = 9 adverse events, *n =* 6 refusal). S-1 administration was halted because of tumor recurrence or other non-S-1-related complications for 3 (8%) arm A (*n* = 1 tumor recurrence, *n* = 2 non-S-1-related complications) and 7 (18%) arm B patients (*n* = 4 tumor recurrence, *n* = 3 other non-S-1-related complications). With exclusion of the censored cases (tumor recurrence and non-S-1-related complications), the completion rates were 80% of arm A and 51% of arm B patients.

**Table 2:** Drug compliance of each course

Arm A (*n* = 38)			Arm B (*n* = 40)		
Course no.	No. of patients completing the course	Reason for discontinuation	Course no.	No. of patients completing the course	Reason for discontinuation
1	36 (94.7%)	Adverse event (2)	1	34 (85.0%)	Patient refusal (3) Adverse event (3)
			2	33 (82.5%)	Patient refusal
2	34 (89.5%)	Adverse event (2)	3	31 (77.5%)	Patient refusal Adverse event
			4	29 (72.5%)	Recurrence Changing hospital
3	34 (89.5%)		5	28 (70.0%)	Adverse event
			6	27 (67.5%)	Patient refusal
4	31 (81.6%)	Adverse event (2) Recurrence	7	27 (67.5%)	
			8	25 (62.5%)	Adverse event Unrelated death
5	31 (81.6%)		9	23 (57.5%)	Recurrence (2)
			10	22 (55.0%)	Adverse event
6	31 (81.6%)		11	21 (52.5%)	Adverse event
			12	19 (47.5%)	Adverse event Recurrence
7	30 (78.9%)	Unrelated death	13	18 (45.0%)	Changing hospital
			14	18 (45.0%)	
8	28 (73.7%)	Patient refusal Changing hospital	15	18 (45.0%)	
			16	18 (45.0%)

**Table 3:** Feasibility of S-1 administered by 2 schedules

	Arm A (*n* = 38)	Arm B (*n* = 40)	*p*
Completion rate	73.7% (95% CI: 58.0%–85.0%)	45.0% (95% CI: 30.7%–60.2%)	0.01
Relative dose intensity	77.2% (95% CI: 66.8%–87.5%)	58.4% (95% CI: 47.3%–69.6%)	0.01

Arm A: 4 weeks of oral S-1 and a 2-week rest over 12 months; arm B: 2 weeks of S-1 and a 1-week rest over 12 months; *CI* confidence interval; *p* value for the completion rate was calculated by the chi-square test. *p* value for the relative dose intensity was calculated with the use of the Student t-test

The averages of the relative dose intensity over 12 months were 77.2% (95% CI: 68.2%–86.2%) in arm A and 58.4% (95% CI: 47.3%–69.6%) in arm B patients (*p* = 0.01, Table 3).

### Adverse events

Drug-related adverse events are listed in Table 4. The primary adverse events were hematological, gastrointestinal, and cutaneous signs and symptoms. Adverse events were recorded for 38 (100%) of arm A patients (grade 1/2 in 89% and grade 3 in 11%) and 39 (98%) of arm B patients (grade 1/2 in 93% and grade 3 in 5%; *p* = 0.42). Severe grade 3 adverse events were observed in 4 (11%) arm A patients (elevated bilirubin, neutropenia, and rash) and in 2 (5%) arm B patients (anorexia and nausea, *p* = 0.43). Elevated bilirubin, AST, ALT, and alkaline phosphatase levels were more frequent in arm A than in arm B patients (*p* = 0.01, <0.01, 0.01, <0.01, respectively). Two patients, 1 each in arm A and B, died during the drug administration period, although the causes death were unknown and were not considered to be related to S-1 administration.

**Table 4:** Drug-related adverse events of S-1 administered by 2 schedules

	Arm A (*n* = 38)				Arm B (*n* = 40)				*p*
	G1/2		G3		G1/2		G3		
	*n*	(%)	*n*	(%)	*n*	(%)	*n*	(%)	
*Hematological*	31	(82)	1	(3)	26	(65)	0	(0)	0.10
Neutropenia	9	(24)	1	(3)	8	(20)	0	(0)	0.36
Thrombocytopenia	12	(32)	0	(0)	11	(28)	0	(0)	0.81
Anemia	27	(71)	0	(0)	23	(58)	0	(0)	0.23
Leukopenia	17	(45)	0	(0)	15	(38)	0	(0)	0.65
*Non-hematological*	35	(92)	3	(8)	35	(88)	2	(5)	0.21
Elevation of Bilirubin	24	(63)	2	(5)	15	(38)	0	(0)	0.01
Elevation of ALP	16	(42)	0	(0)	6	(15)	0	(0)	<0.01
Elevation of AST	16	(42)	0	(0)	5	(13)	0	(0)	<0.01
Elevation of ALT	15	(39)	0	(0)	3	(8)	0	(0)	0.01
Rash	6	(16)	1	(3)	9	(23)	0	(0)	0.45
Anorexia	14	(37)	0	(0)	12	(30)	2	(5)	0.34
Nausea	15	(39)	0	(0)	14	(35)	1	(3)	0.59
Elevation of BUN	1	(3)	0	(0)	1	(3)	0	(0)	1.00
Elevation of Creatinin	1	(3)	0	(0)	5	(13)	0	(0)	0.20
Pigmentation	12	(32)	0	(0)	15	(38)	0	(0)	0.63
Diarrhea	12	(32)	0	(0)	9	(23)	0	(0)	0.44
General fatigue	6	(16)	0	(0)	10	(25)	0	(0)	0.40
Decline in PS	4	(11)	0	(0)	6	(15)	0	(0)	0.74
Vomiting	4	(11)	0	(0)	3	(8)	0	(0)	0.71
Aphthous stomatitis	3	(8)	0	(0)	6	(15)	0	(0)	0.48
Nervous system disorder	2	(5)	0	(0)	1	(3)	0	(0)	0.61
Edema	2	(5)	0	(0)	2	(5)	0	(0)	1.00
Infection	1	(3)	0	(0)	1	(3)	0	(0)	1.00
Others	6*	(16)	0	(0)	11**	(28)	0	(0)	0.19
Total	34	(89)	4	(11)	37	(93)	2	(5)	0.42

Arm A: 4 weeks of oral S-1 and a 2-week rest over 12 months; arm B: 2 weeks of S-1 and a 1-week rest over 12 months; *ALP* alkaline phosphatase, *AST* aspartate aminotransferase, *ALT* alanine transaminase, *BUN* blood urea nitrogen, *PS* performance status; * = dizziness (1), urticaria (1), lacrimation (1), ileus (1), finger cyanosis (1), nasal bleeding (1) and dyspnea (1); ** = nasal bleeding (4), taste disorder (2), dizziness (1), fever (1), dry skin (1), finger bleeding (1), lacrimation (1), cutaneous pruritus (1), blurred vision (1); *p* values were calculated with the use of the chi-square test. The total number of patients of each arm was used as a denominator when calculating category-specific percentages in the table

### Disease-free survival and recurrence

**Fig. 1:**
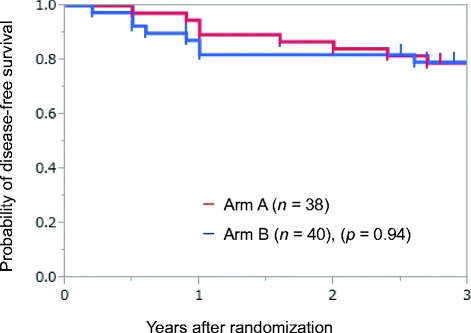
Disease-free survival rate in each arm; 3-year disease-free survival rates were 79.0% in arm A and 79.3% in arm B (*p* = 0.94, log-rank test)

The median follow-up time was 64 months (range 6–113 months). The 3-year DFS rates of arm A and arm B patients were 79.0% and 79.3%, respectively (*p* = 0.94, Fig. 1). A total of 9 (23.7%) arm A and 8 (20.0%) arm B patients relapsed within 3 years. Locoregional recurrence was predominant in both arms; 6 of 9 relapsed arm A and 5 of 8 relapsed arm B patients. The locoregional recurrences in arm A patients were lung metastases (*n* = 4), hilar lymph node metastasis (*n* = 1) and carcinomatous pleurisy (*n* = 1). The locoregional recurrences in arm B patients were lung (*n* = 2), mediastinal lymph nodes (*n* = 1) and carcinomatous pleurisy (*n* = 1). The distant relapses in arm A patients were brain metastasis (*n* = 2) and supraclavicular lymph node metastasis (*n* = 1) and in arm B patients were bone metastases (*n* = 2) and brain metastasis (*n* = 1).

## Discussion

To the best of our knowledge, this is the first multicenter randomized clinical trial that compared the feasibility of 2 S-1 administration schedules in a long-term adjuvant setting after curative surgery. This study showed that the shorter schedule of 2-weeks of S-1 administration and a 1-week rest period resulted in less toxicity than the conventional schedule of 4-weeks of S-1 followed by 2-weeks rest. But the superiority of the completion rate and relative dose intensity of the shorter schedule could not be confirmed in the present study. Before this study was performed, we expected that the shorter administration schedule would be more feasible with less toxicity. Because adverse events associated with S-1 tend to be observed starting 2 to 3 weeks after initiation of S-1 treatment, a shorter administration schedule was thought to be advantageous. However, the results showed that the shorter administration schedule was not superior for the completion rate and the relative total administration dose. Toxicity showed no significant difference among the shorter schedule and the conventional schedule, except for grade 1–3 elevation of bilirubin. The reasons for stopping S-1 for patients taking it according to the shorter schedule included a 23% adverse event rate and a 17% patient refusal rate, and the reasons for stopping S-1 for patients taking it according to the conventional schedule included a 16% adverse event rate and a 3% patient refusal rate. Patient refusal might account for the lower feasibility of the shorter administration schedule.

Patient compliance is reported to be a problem in trials of adjuvant chemotherapy [4]. In trials of cisplatin-based chemotherapy that was scheduled to be administered in 3 or 4 cycles postoperatively, only 50%–74% of the patients completed the planned treatment [14–18]. Even with the infrequent and usually mild adverse reactions of oral UFT, only 61% of patients completed the 2-year course [4]. Compliance in trials of adjuvant chemotherapy may not be related to the severity of adverse events [4]. A feasibility study of adjuvant S-1 for gastric cancer had a completion rate of 60.7%, with a high rate of patient refusal due to adverse reactions, especially after the first course (anorexia) [19]. Based on the results of the Adjuvant Chemotherapy Trial of S-1 for Gastric Cancer [8], patients were estimated to refuse S-1 administration even with grade 1 or 2 digestive system adverse events [20]. In a feasibility study of adjuvant S-1 for elderly patients with NSCLC, both the patients and their physicians were speculated to be less willing to tolerate even modest degrees of toxicity, particularly because the benefits of adjuvant chemotherapy were unproven [10]. Those patients “less willing to tolerate even a modest degree of toxicity” would negatively affect the feasibility of long-term administration. The shorter S-1 administration schedule in our study had twice the number of administration cycles, and although each cycle consisted of half the conventional administration dose, the increased number of cycles might have led to increased opportunities of thinking about refusal.

The completion rates of adjuvant S-1 administered with a conventional schedule for patients with gastric cancer have been reported to be 78% for 6 months [8] and 61%–66% for 1 year [8, 19]. The completion rates of S-1 for patients with lung cancer have been reported to be 61%–71% for 6 months with the shorter schedule [10, 11] and 50%–72% for 1 year with the conventional schedule [12, 13]. In our study, the completion rates for 1 year were 74% with the conventional schedule and 45% with the shorter schedule. With exclusion of the censored cases, the completion rates were 80% and 51%, respectively. Our study found a relatively better completion rate over 1 year with the conventional administration schedule than the other studies. Based on the assumption that patients will refuse to continue S-1 with even grade 1 or 2 digestive system adverse events [20], the prophylactic use of lafutidine, a histamine H2 receptor antagonist, to reduce the occurrence of gastrointestinal toxicities might improve patient compliance. There has been a recent report on the efficacy of lafutidine for reducing gastrointestinal toxicity during adjuvant S-1 chemotherapy for patients with gastric cancer [21]. The rate of patients requiring a dose reduction or interruption of S-1 treatment was significantly lower in the arm receiving S-1 plus lafutidine than in S-1 alone (30% vs. 83%, respectively).

The 3-year DFS rates were 79.0% for the conventional S-1 schedule and 79.3% for the shorter schedule, which were not significantly different. In this study, 78% of the patients had stage IB disease, and we believe that the 3-year DFS of 79.0%–79.3% is acceptable. Tsuchiya et al. [12] reported comparable results for patients with curatively resected stage IB-IIIA NSCLC who were treated by adjuvant S-1 administration for 1-year. The 3-year DFS was 69.4% and the 3-year survival rate was 87.7%. The 2004 Japanese Lung Cancer Registry Study of 11,663 surgical cases (adjuvant therapy was performed in 2903 [24.9%] cases and induction chemotherapy in 518 [4.4%] cases) [22] found 3-year survival rates of 79.1% for patients with p-stage IB and 53.7% for patients with p-stage IIIA disease [23]. Our study should continue to collect additional follow-up survival data, because adjuvant UFT showed relatively delayed survival benefit after 4 years of follow up [6] and the adjuvant S-1 might also show the similar survival benefit. UFT and its metabolites were reported to have antiangiogenic activity [24], which is considered to be one of the mechanisms for its long-term effectiveness. S-1 shows promise as an adjuvant chemotherapy that is suitable for long-term administration to outpatient administration, and has shown higher antitumor activity than UFT. A phase II trial of S-1 monotherapy as first line treatment for patients with advanced NSCLC found a response rate of 22% [25]. A randomized phase III trial demonstrated that S-1 plus carboplatin for patients with advanced NSCLC was noninferior for overall survival, compared with paclitaxel plus carboplatin [26], regardless of tumor histology [27]. Another randomized phase III trial demonstrated that S-1 plus cisplatin for patients with advanced NSCLC was noninferior for overall survival, compared with docetaxel plus cisplatin [28]. Therefore S-1 is becoming one of the standard chemotherapy regimens for patients with NSCLC in Japan.

Major limitations of our randomized controlled trial are diagnostic bias of the endpoints and the small study sample size. Our study was open-label trial and the doctors and patients already knew which regimen they were allocated. The open-label trial always suffered from the diagnostic bias and our results was not the exception. Though feasibility (completion rate) is rather objective than the toxicity, we should understand that both measures suffered the diagnostic bias in our study. The second issue of our study is its small sample size. The initiation of this study is April 2005 and patients’ enrollment took 7 years to reach 80 patients. The main reason for this slow enrollment was the emergence of new treatment, adjuvant platina doublet for pathological stage II and IIIA. This treatment was stated as the standard in the guideline for lung cancer in Japan. The introduction of this new treatment affected our enrollment and the motivation of doctors and patients may be different according to disease stage. The completion rates over 12 months among pathological stage or among institutions showed no differences.

## Conclusions

The superiority of feasibility of the shorter schedule was not recognized in the present study. The conventional schedule showed higher completion rates over 12 months (*p* = 0.01) and relative dose intensity of S-1 (*p* = 0.01). Toxicity showed no significant difference among the shorter schedule and the conventional schedule, except for grade 1–3 elevation of bilirubin.

## Abbreviations

BSA: body surface area
CI: confidence interval
CTCAE: Common Terminology Criteria for Adverse Events
DFS: disease-free survival
ECOG: Eastern Cooperative Oncology Group
NSCLC: non–small cell lung cancer
PS: performance status
